# Targeting YOD1 by RNA Interference Inhibits Proliferation and Migration of Human Oral Keratinocytes through Transforming Growth Factor-*β*3 Signaling Pathway

**DOI:** 10.1155/2018/6254308

**Published:** 2018-09-13

**Authors:** Xiao-Long Zhou, Gang Chen, Meng-Xue Li, Heng-Xue Wang, Jia-Wei Hong, Jun-Yu Shen, Qi Wang, Xing Ge, Zhen Ding, Li-Chun Xu

**Affiliations:** School of Public Health, Xuzhou Medical University, 209 Tong-Shan Road, Xuzhou, Jiangsu 221004, China

## Abstract

**Objective:**

We have identified a gene YOD1 encoding deubiquitinating enzyme (DUB) responsible for nonsyndromic cleft lip with or without cleft palate (NSCL/P). We aimed to determine the effects of YOD1 RNA interference (RNAi) on cell proliferation and migration, playing an important role in lip and palate formation, and to clarify whether the mechanisms involved TGF-*β*3 signaling associated with NSCL/P.

**Methods:**

RNAi was applied to construct vectors expressing YOD1 small interference RNAs (siRNAs). The vectors were transfected into the human oral keratinocytes (HOK) cells. The cell proliferation and migration were evaluated by the cell counting kit-8 (CCK-8) assay and wound healing assay, respectively. The mRNA levels were detected by quantitative real-time reverse transcription-polymerase chain reaction (qRT-PCR). The protein levels were investigated by western blotting.

**Results:**

The proliferation of YOD1 siRNA-transfected HOK cells was remarkably inhibited. The migration rate was significantly decreased in the YOD1 siRNA-transfected HOK cells. The TGF-*β*3 mRNA and protein levels were decreased significantly by siRNA-mediated knockdown of YOD1. YOD1 RNAi reduced the phosphor-Smad2/3 levels significantly.

**Conclusions:**

YOD1 RNAi may inhibit cell proliferation and migration associated with the pathogenesis of NSCL/P through TGF-*β*3 signaling. The study indicates a novel role of YOD1 in regulating TGF-*β*3 signaling to affect cell proliferation and migration resulting in NSCL/P.

## 1. Introduction

As one of the most common birth defects, nonsyndromic cleft lip with or without cleft palate (NSCL/P) is a complex congenital malformation with a prevalence of 1-2/1 000 births in all populations worldwide [1]. NSCL/P influences suckling, swallowing, and the development of language and hearing. Individuals with NSCL/P are also associated with an increased risk of cancers [2] and an elevated overall mortality into adulthood [3]. NSCL/P increases economic and psychosocial burdens on the subjects, their families, and the society [4].

The etiology of NSCL/P is considered to be due to both environmental and genetic factors. Many studies suggest that genetic factors play crucial roles in the pathogenesis of NSCL/P [5–9]. A large number of genes are suspected to be involved in NSCL/P probably to affect the embryonic development and differentiation leading to the formation of the facial structures [10–14]. Although the etiology of NSCL/P has been widely investigated [1, 6, 15], there is inconsistency across studies. Specific genes and mechanisms responsible for NSCL/P have not been fully understood.

In our previous study, we have identified the 54G/T single nucleotide polymorphisms (SNPs) in one deubiquitinating enzyme (DUB) gene YOD1 through whole-exome sequencing (WES) [16]. Therefore, we speculate that YOD1 may be a novel susceptibility gene responsible for NSCL/P. However, the biological mechanisms of YOD1 involved in NSCL/P pathogenesis are poorly characterized and need to be clarified.

The transforming growth factor-*β* (TGF-*β*) signaling pathway mediated by TGF-*β*, especially by TGF-*β*3, plays an important role in the embryonic development and NSCL/P pathogenesis [17, 18]. Studies have shown that TGF-*β* is expressed in many cell types including epithelial cells and controls proliferation, motility, and differentiation throughout embryogenesis and adult life [19]. TGF-*β* signaling is initiated when ligands bind to their cognate receptors such as type I (T*β*RI) and type II (T*β*RII, also known as ALK5, activin receptor-like kinase 5). The Smad proteins including the receptor-regulated Smads (R-Smads) are the intracellular transducers [20]. Upon ligands binding, the T*β*RII is phosphorylated and activates the T*β*RI. Activated T*β*RI phosphorylates the R-Smads including Smad2 and Smad3 to induce the formation of Smad2/3 and co-mediator Smad (co-Smad) Smad4 complex and nuclear translocation. In the cell nucleus, the complex binds DNA along with the nuclear cofactors to regulate gene expression finally.

TGF-*β* signaling pathway can be regulated by DUBs. UCH37, a member of DUBs, can deubiquitinate and stabilize the T*β*RI which is targeted for ubiquitylation-mediated degradation. Overexpression of UCH37 upregulates TGF-*β*-dependent transcription which is reversed in cells subject to RNAi-mediated knockdown of UCH37 [21]. USP4, also a DUB, can directly interact with T*β*RI and control the receptor levels at the plasma membrane to enhance TGF-*β* signaling [22]. USP15, also a DUB, is required for TGF-*β* and BMP responses in mammalian cells and Xenopus embryos and stabilizes SMAD2/3 to enhance TGF-*β* signaling [23]. OTUB1, a member of ovarian tumor domain protease (OTU) family of DUBs, is confirmed to play an important role in the TGF-*β*-mediated gene transcription and cellular migration and enhance TGF-*β* signaling by inhibiting the ubiquitylation and degradation of phospho-SMAD2/3 [24]. Consequently, DUBs may stabilize TGF-*β* receptor and Smads to enhance TGF-*β* signaling [25].

YOD1, also known as OtuD2 or DUBA8, is a member of DUBs of OTU family [26]. YOD1 is related to various development processes [27]. Therefore, we speculate that YOD1 may also be involved in the TGF-*β* signaling pathway due to the activity of DUB, which plays a role in NSCL/P progression through affecting the embryonic development and differentiation. However, little is known about the effects of YOD1 on TGF-*β*3 signaling.

In this study, we exploited RNA interference (RNAi) targeting YOD1 to downregulate the expression of YOD1. We have shown that knockdown of YOD1 by RNAi inhibits the proliferation and migration of human oral keratinocytes (HOK) cells. The involved possible mechanisms were also explored. The results have shown that RNAi of YOD1 reduces TGF-*β*3 and phosphor-Smad2/3 expression. YOD1 serves as an important gene affecting cell proliferation and migration through regulating TGF-*β*3 signaling associated with NSCL/P.

## 2. Material and Methods

### 2.1. Cell Culture

The HOK cells were provided by Shanghai Jiaotong University and were cultured in RPMI 1640 medium containing 15% fetal bovine serum (FBS) (Life Technologies). Cells were grown in a humidified incubator at 37°C and 5% CO_2_ for subculture.

### 2.2. YOD1 Small Interfering RNA (siRNA) and siRNA Transfections

Three double-stranded siRNAs oligonucleotides against YOD1 were designed and chemically synthesized by Guangzhou RiboBio Co., Ltd, China. Then the siRNA (5'-GAGACAGGCCAUACCAACUdTdT-3'; 3'-dTdTCUCUGUCCGGUAUGGUU GA-5') with the highest inhibition rate against YOD1 was selected for the subsequent experiments. Briefly, 24 h prior to transfection, HOK cells were seeded in 6-well plates, with 2.5× 10^5^ cells per well, corresponding to a density of 40%-60% at the time of transfection. Four groups were applied. In siRNA group, cells were transfected with siRNA at a dose of 100nmol/L. In negative control (NC) group, cells were transfected with NC siRNA (TM, Guangzhou RiboBio Co., Ltd, China) which had no effect on YOD1. In Lipo2000 group, cells were treated with Lipofectamine 2000 (Invitrogen, USA) alone without siRNA. In blank control group, the cells were untransfected. All transfections were performed in triplicate for each time point. At different times after the beginning of the transfection period, all cells were harvested and the following assays were performed. The medium was replaced with normal medium without antibiotics after 6 h transfection. Cells were treated and the following assays were performed 48 h later.

### 2.3. Quantitative Real-Time Reverse Transcription-Polymerase Chain Reaction (qRT-PCR)

The TRIzol reagent (Life Technologies, USA) was used to extract the total RNA of HOK cells. cDNAs were synthesized using Reverse Transcription Kit (TaKaRa, JP). The qRT-PCR analyses for target genes were processed with the SYBR Green PCR Master mix (TaKaRa) on an ABI 7500 real-time PCR system (Applied Biosystems, Foster City, CA), and GAPDH was used for standardization. The following primers were used for PCR: YOD1, sense 5'-CTTCCCTGATCCAGAT ACACCTCCT-3', antisense 5'-TCCCTTGCTTCTGCTTGTCCAGTT-3'; TGF-*β*3, sense 5'-CCTCTACATTGACTTCCGACA-3', antisense 5'-GGCAGATGCTTCA GGGTTC-3'; GAPDH, sense 5'-CATGTGGGCCATGAGGTCCACCAC-3', antisense 5'- GGGAAGCTCACTGGCATGGCCTTCC-3'. The data were analyzed with the 2ΔΔCT method as ΔΔCT = (CT_Target1_ − CT_GAPDH_)−(CT_Target2_ − CT_GAPDH_).

### 2.4. Western Blot

HOK cells were harvested and then lysed in a lysis buffer supplement with protease inhibitors (Beyotime Institute of Biotechnology, Haimen, China) and phosphatase inhibitors (Nanjing KeyGEN Biotech. Co., Ltd, China) for extraction of total cellular protein. The protein quantification was obtained by bicinchoninic acid protein assay kit (Beyotime Institute of Biotechnology). For western blot analyses, 20-30 *μ*g of total extracted proteins were injected into each lane before SDS-PAGE. Following transfer to polyvinylidene fluoride membranes, polyclonal anti-YOD1 (Proteintech, USA), anti-TGF-*β*3 (Proteintech), anti-Smad2/3 (Cell Signaling, Danvers, MA), and anti-phospho-Smad2/3 (Cell Signaling) were used for the detection of the protein expression levels. The expression of beta-tubulin (Cell Signaling) was used as an internal reference for protein loading.

### 2.5. Cell Counting Kit-8 (CCK-8) Assay

Each group of HOK cells following transfection in the logarithmic growth phase was collected. Then the viable cells in each group were counted, and cells were inoculated in 96-well plates with each group set in five parallel wells. Total 5000 cells were seeded in each well. Following 0, 24, 48, 72, and 96 h inoculation, 10 *μ*l CCK-8 solution (Dojindo, Kumamoto, Japan) was added to each well and then cultured in an incubator at 37°C for 2 h. A microplate reader was used to detect the optical density (OD) values at 450 nm wavelength, and a blank well was set as zero. At last, the cell growth curves were drawn.

### 2.6. Wound Healing Assay

After transfection for 48 h, the HOK cells were collected and inoculated in 12-well plates (5×10^5^/well). A line-shaped scratch using a sterile was applied by 10 *μ*l pipette tip at the surface of the cell monolayer after the cells merged into a monolayer. Phosphate Buffered Saline (PBS) was used for washing three times. The width between the borders of the scratches was observed and images were obtained in each group at 0, 12, and 24 h with an inverted microscopy equipped with a digital camera (Olympus Corporation, Japan).

### 2.7. Statistical Analysis

Statistical analysis was processed with SPSS 16.0 statistical software (SPSS, Inc., Chicago, USA). The experiments above were performed at least in triplicate. Quantitative data were presented as the mean ± SD. Multiple comparisons were made using one-way analysis of variance (ANOVA), and Dunnett's* t*-test was used for multiple comparisons with the control. The data of CCK-8 were analyzed by using the repeated measure data analysis of variance.* P* values less than 0.05 were considered statistically significant.

## 3. Results

### 3.1. Silencing of the YOD1 Gene by RNAi

**Figure 1:**
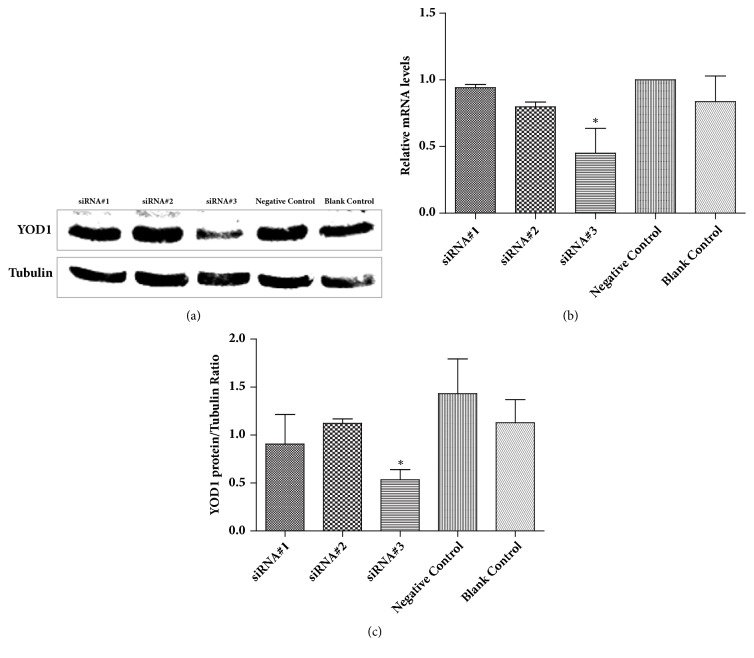
RNAi knockdown of YOD1 in HOK cells. (a) The protein level of YOD1 in HOK cells was detected by western blot. (b) qRT-PCR was used for detection the effect of YOD1 RNAi on the mRNA expression of YOD1. (c) Quantitative densitometry evaluation of YOD1 was shown. Data were expressed as mean ± SD from three separate experiments. Statistical analyses were performed using the t-test and one-way ANOVA. *∗P* < 0.05 versus the control groups.

To verify whether the YOD1 gene was silenced by designed siRNA, we determined the effects of the three chemosynthetic siRNAs (#1, #2, and #3) in HOK cells after transient transfection. The three siRNAs against YOD1 reduced mRNA and protein expression, and cells transfected with siRNA#3 showed the most significantly reduced mRNA and protein levels of YOD1 (Figure 1). Therefore, the siRNA#3 was selected to study the function of YOD1.

### 3.2. Inhibition of Cell Proliferation by YOD1 siRNA

**Figure 2:**
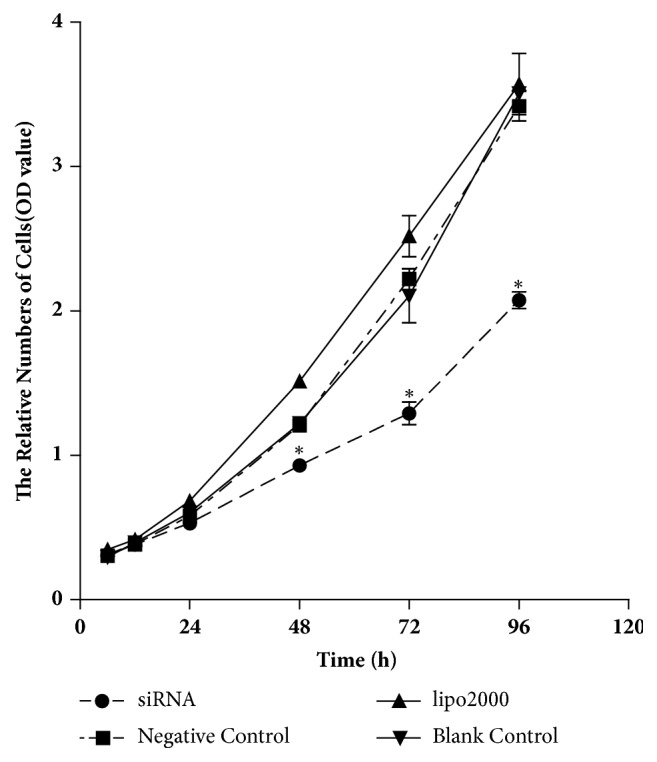
Downregulation of YOD1 expression inhibited the proliferation ability of HOK cells. HOK cells transfected with negative control and YOD1 siRNA were used for CCK8 assay. Cell viability in YOD1 siRNA-transfected cells was significantly reduced compared with corresponding controls. Shown are the mean ± SD values from three separate experiments of each group. *∗P* < 0.05 versus the control groups.

We used the CCK-8 assay to assess the effect of YOD1 siRNA on proliferation of HOK cells cultured in 96-well plates. After 48 h of transfection, the YOD1 siRNA-transfected cells showed a decrease in proliferation compared to those of negative siRNA-transfected cells and the blank control cells (Figure 2).

### 3.3. Inhibition of Cell Migration by YOD1 siRNA

**Figure 3:**
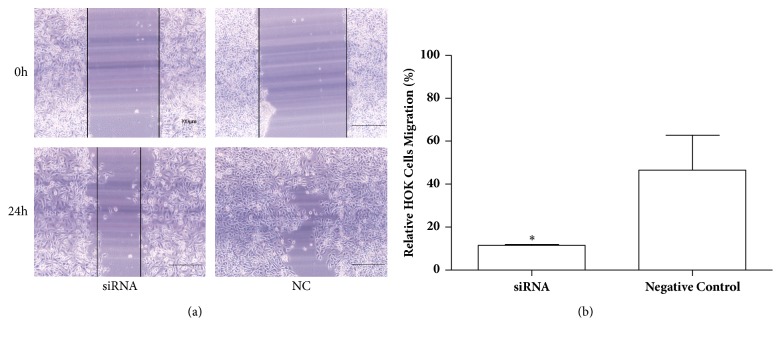
Knockdown of YOD1 inhibited HOK cells migration determined by the scratch wound assay. HOK cells were treated with siRNA of YOD1 and negative siRNA, respectively, for 48h and serum starved for 24h, and then the ability of migration was detected by the scratch wound assay. Representative photomicrographs of HOK cells migration were taken and the relative distances of migration were measured by inverted fluorescence microscope. (a) The typical photomicrographs of HOK cells migration. (b) The results showed that the relative mobility of cells in negative control group was significantly higher than that in siRNA group. Data were expressed as mean ± SD from three separate experiments. *∗P* < 0.05 versus the negative control. (Original magnification 100×.)

We studied the influence of YOD1 knockdown on the HOK cells migration capacity by the wound scratch assay. After 48 h of transfection, migration of cells transfected with YOD1 siRNA was reduced significantly as compared with the negative siRNA-transfected cells and the blank control cells (Figure 3).

### 3.4. Effects of YOD1 siRNA on TGF-*β*3 Signaling Pathway

**Figure 4:**
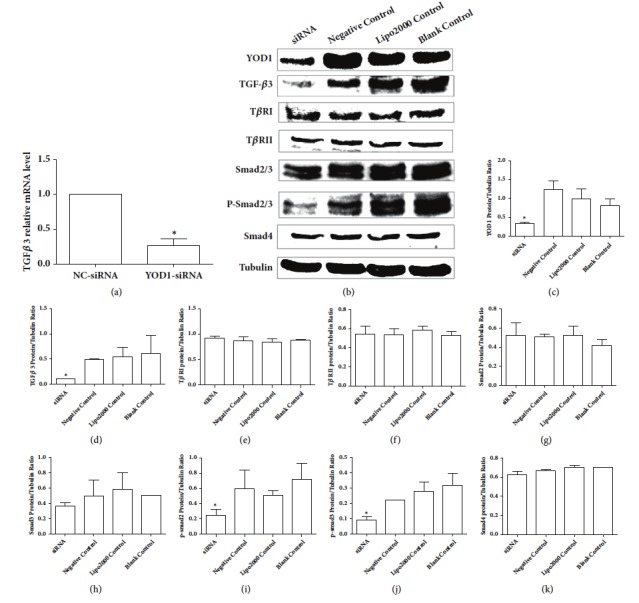
YOD1 played important roles by affecting the activation of TGF-*β*3/Smad signaling pathway. (a) qRT-PCR showed that the mRNA expression of TGF-*β*3 in the HOK cells transfected with YOD1-specific siRNA was decreased compared with the control groups. Data are shown as mean ± SD of three replicate experiments from each group. *∗P* < 0.05 versus the negative control. (b) Protein levels of YOD1, TGF-*β*3, T*β*RI, T*β*RII, total Smad2/3, p-Smad2/3 and Smad4 were determined by western blot analysis. Representative western blots are shown. Densitometric analysis of western blots was used to quantify protein expression. Quantitative densitometry evaluation of (c) YOD1, (d) TGF-*β*3, (e) T*β*RI, (f) T*β*RII, (g) Smad2, (h) Smad3, (i) p-Smad2, (j) p-Smad3, and (k) Smad4 was shown. Results are presented as fold-change compared to control. Blots were normalized with tubulin for total extracts. Data are shown as mean ± SD of three replicate experiments from each group. *∗P* < 0.05 versus the negative control.

In order to further explore whether the TGF-*β*3 signaling pathway was affected by YOD1, the expression level of mRNA of TGF-*β*3 and the protein expression levels of TGF-*β*3, T*β*RI, T*β*RII, total Smad2/3, phosphor-Smad2/3, and Smad4 were examined. Compared to the negative control cells, the mRNA and protein levels of TGF-*β*3 were remarkably decreased in the cells of YOD1 gene silencing (Figures 4(a) and 4(b)). The protein levels of phosphor-Smad2/3 were significantly reduced in the HOK cells transfected with YOD1 siRNA as compared with the negative controls (Figures 4(b), 4(i), and 4(k)). However, the protein levels of T*β*RI, T*β*RII, total Smad2/3, and Smad4 were not affected by YOD1 siRNA (Figures 4(b), 4(e), 4(f), 4(g), 4(h), and 4(k)). These findings suggested that the TGF-*β* signaling pathway was affected by YOD1 siRNA (Figure 4).

## 4. Discussion

In the present study, we have shown that knockdown of YOD1 by siRNA inhibited proliferation and migration of HOK cells in* vitro*. Therefore, YOD1 may play an important role in regulating the abilities of cell proliferation and migration. Furthermore, we have found that YOD1 siRNA decreases the mRNA levels of TGF-*β*3 and protein levels of phosphor-Smad2/3. It is indicated the TGF-*β*3 signaling pathway may be affected by YOD1 siRNA. We provide evidence for the first time that YOD1 contributes to changing the abilities of cell proliferation and migration regulated by TGF-*β*3 signaling pathway, which may be involved in the pathogenesis of NSCL/P.

We explored the effects of YOD1 siRNA on the proliferation and migration of HOK cells. The CCK-8 assay showed that knockdown of YOD1 by siRNA inhibited the HOK cells proliferation. Furthermore, we found that YOD1 siRNA significantly inhibited migration of HOK cells. Our previous study has identified YOD1 as a possible susceptibility gene for NSCL/P [28]. Cell proliferation and migration have been shown to play an important role in the formation of the facial structures [29, 30]. Therefore, our data suggest that the inhibition of cell proliferation and migration caused by YOD1 siRNA may be associated with the occurrence and development of NSCL/P. However, a recent study showed that downregulation of YOD1 may promote cell proliferation in cervical cancer cells [28]. The inconsistent results may be due to the different cell types. Further studies are needed to explore the effects of YOD1 on proliferation and migration of more different cells.

The molecular mechanisms of how YOD1 affects the cell proliferation and migration need to be fully clarified. Studies have shown that TGF-*β*3 plays a crucial role in the process of lip and palate tissue development during the embryonic period [31, 32]. TGF-*β*3 has been proposed as a key component in palatal fusion [33], and the TGF-*β*3 signaling pathway mediated by TGF-*β*3 controls a lot of cellular functions during embryogenesis, including differentiation, proliferation, and migration [34]. It is possible that TGF-*β*3 signaling may be related to cell proliferation and migration modulating by YOD1. The purpose of this study is to detect whether the TGF-*β*3 signaling pathway is involved in cell proliferation and migration affected by YOD1 siRNA in HOK cells. The present study indicated that the mRNA and protein expression levels of TGF-*β*3 were remarkably decreased by YOD1 siRNA. We suggest that TGF-*β*3 is associated with inhibition of cell proliferation and migration affected by YOD1 siRNA. TGF-*β*3 signaling is activated by assembling receptor complex that activates Smad transcription factors [35]. TGF-*β*3 has a high affinity for T*β*RII and binds to the receptor to activate it. Subsequently, the receptor T*β*RI is recruited into the complex and is activated. Thus, TGF-*β*3 stimulation brings together heterotetrameric active receptor complex to result in phosphorylation-dependent activation of the Smad proteins and downstream signaling cascades. Therefore, TGF-*β*3 plays a crucial role in controlling cellular function through initiating the TGF-*β*3 signaling. The inhibition of TGF-*β*3 signaling may lead to NSCL/P through suppressing cell proliferation and migration.

TGF-*β*3 signaling is mediated through Smad proteins, which translocate into the nucleus to act as transcription factors for target genes [36]. The activated receptor complex recruits the R-Smad including Smad2 and Smad3 and are specifically phosphorylated at the carboxyl terminus by T*β*RI. The phosphorylation events result in dissociation of R-Smad from the receptor complex, association with the co-Smad Smad4, nuclear translocation, and subsequent transcriptional activation or repression. Therefore, the phosphorylation of Smad2 and Smad3 is critical for TGF-*β*3 signaling activation. The results of our study have shown that silencing of YOD1 by RNAi in HOK cells inhibits protein level of phosphor-Smad2/3 which may be associated with inhibition of TGF-*β*3 expression by YOD1 siRNA.

Our data suggest that YOD1 siRNA will reduce the expression levels of TGF-*β*3 and phospho-Smad2/3. Silencing of YOD1 by RNAi has been shown to downregulate TGF-*β* signaling. Therefore, YOD1 may be involved in the TGF-*β* signaling pathway. Studies have shown that DUBs play an important role in TGF-*β*-mediated gene transcription and cell migration [24]. YOD1 belongs to OTU family of DUBs [37]. YOD1 siRNA may enhance the ubiquitylation of TGF-*β*3 and phospho-Smad2/3 and then result in the downregulation of TGF-*β* signaling.

The mechanism of inhibition of cell proliferation and migration caused by YOD1 siRNA may be associated with TGF-*β*3 signaling pathway, which is involved in NSCL/P. One study has discovered that inactivation of TGF-*β*3 leads to cleft palate defect [38]. Many studies have demonstrated that TGF-*β* signaling plays a pivotal role in palate fusion [17, 18, 39]. Therefore, our results suggest that the repression of TGF-*β*3-mediated transcriptional activity mediated by YOD1 RNAi may inhibit cell proliferation and migration to induce NSCL/P.

In conclusion, we have shown that the downregulation of YOD1 using RNAi inhibits proliferation and migration of HOK cells, which involve inhibiting the TGF-*β*3 signaling pathway. YOD1 RNAi inhibits TGF-*β*3 signaling pathway to suppress cell proliferation and migration contributing to NSCL/P.

## Data Availability

The data used to support the findings of this study are available from the corresponding author upon request.
